# Maternal Outcomes Associated with Delayed Cord Clamping in Hypertensive Disorders of Pregnancy: A Cross-Sectional Study

**DOI:** 10.3390/diseases14030107

**Published:** 2026-03-13

**Authors:** Aigerim Turekulova, Nurzhamal Dzhardemaliyeva, Heike Rabe, Mukhtar Kulimbet

**Affiliations:** 1 School of General Medicine, Asfendiyarov Kazakh National Medical University, Almaty 050012, Kazakhstan; 2 International Faculty, Asfendiyarov Kazakh National Medical University, Almaty 050012, Kazakhstan; dzhardemalieva.n@kaznmu.kz; 3 Academic Department of Paediatrics, Brighton and Sussex Medical School, University of Brighton and University of Sussex, Brighton BN1 9PX, UK; 4 Department of Neonatology, University Hospitals Sussex, Brighton BN2 5BE, UK; 5 Department of Research Management, Research Institute of Cardiology and Internal Diseases, Almaty 050000, Kazakhstan

**Keywords:** delayed cord clamping, postpartum hemorrhage, hypertensive disorders of pregnancy, maternal outcomes, preeclampsia

## Abstract

Background/Objectives: Delayed umbilical cord clamping (DCC) is widely recommended for neonatal benefit; however, concerns persist among professionals that DCC may increase the risk of postpartum hemorrhage. There is a higher risk of postpartum hemorrhage in women with hypertensive disorders of pregnancy (HDP). We aimed to evaluate the association between umbilical cord clamping timing and maternal blood loss in term pregnancies, including those complicated by HDP. Methods: We conducted a cross-sectional study of women delivering at three major hospitals in Almaty, Kazakhstan (August 2020–March 2021). The primary outcome was maternal blood loss. Secondary outcomes included hemoglobin (Hb) and red blood cell (RBC) change from pre-delivery to discharge. Multivariable models were adjusted for maternal age, parity and hypertension category. Results: Two hundred and seven women were analyzed (early cord clamping ≤ 60 (ECC) *n* = 21; delayed cord clamping 60–119 s (DCC60s) *n* = 161; delayed cord clamping ≥ 120 s (DCC120s) *n* = 25). Baseline characteristics were similar across groups except for hypertension distribution. Median blood loss did not differ significantly (255–260 mL; *p* = 0.9128). Adjusted models confirmed no association between clamping category and blood loss (RoM: ECC vs. DCC60s 0.97; 95% CI 0.93–1.01; DCC120s vs. DCC60s 1.01; 95% CI 0.96–1.07). Conclusions: Among term births in Almaty, including HDP-affected pregnancies, delayed umbilical cord clamping was not associated with increased maternal blood loss or hematologic decline. These findings indicate that DCC does not appear to increase maternal bleeding risk in high-risk obstetric populations and are broadly in line with current international recommendations. Further prospective research is warranted to evaluate specific subgroups, including severe preeclampsia.

## 1. Introduction

Maternal health is a key global priority, with pregnancy complications remaining a leading cause of morbidity and mortality in women and infants [1]. Hypertensive disorders of pregnancy complicate roughly 5–15% of pregnancies worldwide and are a major contributor to maternal and perinatal morbidity and mortality globally [2] and in Kazakhstan [3]. Women with hypertensive disorders of pregnancy face higher risks of hemorrhage and end-organ damage [4,5]. Addressing preventable causes of maternal illness and death in this context is therefore essential to improving maternal health outcomes globally.

Delayed umbilical cord clamping (DCC) has gained acceptance because it provides neonatal benefits such as higher infant hemoglobin and iron stores and better development [6,7], without clear harms [8]. Professional bodies now recommend clamping the cord at least 30–60 s after birth to allow placental-to-infant blood transfer [9,10,11,12]. Concern has been raised that delaying cord clamping might increase maternal blood loss, especially when placental separation is still underway. In a systematic review [13], it was noted that in 12 studies, staff-level barriers and persistent professional bias towards this method were identified, which hindered its application despite the adoption of protocols. In some studies, although the majority of obstetricians and midwives participating in the surveys held a favorable attitude towards the practice of delayed cord clamping, they reported that the method had not been widely disseminated or applied in their routine practice [14]. However, studies demonstrated no increased risk of postpartum hemorrhage among women with either term or preterm births [15,16]. Some practitioners worry that extended time to clamp the cord could prolong the third stage of labor or delay uterine contraction, potentially raising postpartum hemorrhage risk.

Randomized trials and meta-analyses have consistently found no increase in maternal bleeding associated with DCC in term births [8,16]. However, these data are derived almost exclusively from cohorts of healthy women with uncomplicated pregnancies, and high-risk groups are underrepresented. No large observational study has specifically evaluated how the timing of cord clamping affects maternal blood loss and hematologic changes in a broader cohort of women, including those with hypertension. In particular, little is known about how the timing of cord clamping affects maternal outcomes in pregnancies complicated by hypertensive disorders, despite the fact that such conditions are associated with increased risk of hemorrhage and hematologic derangements [4].

Therefore, the aim of this study was to determine the effects of umbilical cord clamping timing on maternal blood loss, including in women with hypertension. Specifically, we evaluated the association between early versus delayed cord clamping and postpartum blood loss.

## 2. Materials and Methods

### 2.1. Study Design and Setting

We conducted a cross-sectional study of women who delivered from August 2020 to March 2021 at three major hospitals in Almaty, Kazakhstan.

The participating hospitals are high-volume obstetric facilities in Almaty providing 24/7 maternity services. Their daily activity includes admission/triage of laboring women and referrals, management of vaginal and operative deliveries (including cesarean section), routine postpartum monitoring, and neonatal care immediately after birth, supported by round-the-clock obstetric, anesthesia, midwifery, and neonatal teams. Study participants were recruited from routine daily delivery admissions during August 2020–March 2021.

### 2.2. Inclusion and Exclusion Criteria

Inclusion criteria: All women with singleton and full-term pregnancies and documented cord clamping time were eligible.

Exclusion criteria: Women were excluded from the study if the required information was incomplete, if they had obstetric complications likely to confound bleeding assessment (e.g., placenta previa, antepartum hemorrhage, known coagulopathies, or major fetal anomalies), if they had a positive COVID-19 test, or if they were younger than 18 years of age.

**Figure 1:**
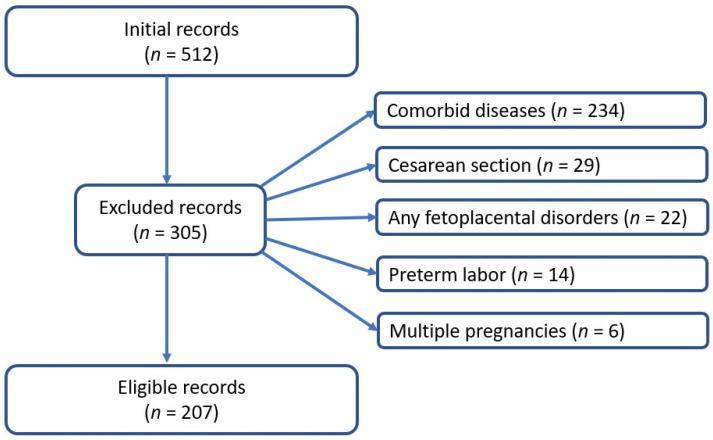
This is a flow chart of eligible participants. Initial data included 512 records. After applying the inclusion and exclusion criteria, 207 records remained.

Figure 1 presents the flow chart of eligible participants. In the final analysis, 207 women were included, of whom 111 were normotensive and 96 had hypertensive disorders of pregnancy.

### 2.3. Participants

Participants were pregnant women who gave birth at 37 weeks of gestation or later.

### 2.4. Data Collection

Data were collected retrospectively from medical records and observation notes of deliveries. The research team did not intervene in clinical decision-making or in the management of labor and delivery. The timing and technique of umbilical cord clamping were determined entirely by the attending obstetrician and the patient according to standard clinical practice. Investigators only recorded the clamping time, maternal and neonatal characteristics, and relevant perinatal outcomes without influencing clinical care in any way.

### 2.5. Cord Clamping Procedure

ECC was performed by clamping the cord as soon as possible after the infant was born, and the time was noted. In contrast, in the DCC60s and DCC120s groups, clamping was done at least 60 s after birth, and the time was noted.

### 2.6. Variables

Outcomes: The primary outcome was maternal blood loss during delivery, expressed in milliliters as recorded in delivery notes [17]. Secondary “derived hematologic” outcomes captured maternal blood change more sensitively than single measures:

- Hemoglobin drop = Hb_index − Hb_discharge (g/L), where Hb_index was the last hemoglobin measured before delivery and Hb_discharge was the first hemoglobin measured before hospital discharge.
- RBC drop = RBC_index − RBC_discharge (×10^12^/L), defined analogously for red blood cell count. These delta values reflect peri-delivery changes in maternal blood indices.

Exposure: The main exposure was the timing of umbilical cord clamping after birth. We recorded clamping time in seconds and categorized it as early (ECC) (0–59 s), delayed (DCC60s) (60–119 s; reference group), and late delayed (DCC120s) (≥120 s). In adjusted models, we also treated cord time as a continuous variable [18,19,20].

Covariates: Pre-delivery confounders included maternal age (years), parity (nulliparous vs. multiparous), gestational age at delivery (weeks), and hypertension category (normotensive, gestational hypertension, chronic hypertension, or preeclampsia) [21,22,23]. These factors were chosen a priori for their known association with both pregnancy management and risk of hemorrhage.

### 2.7. Ethics

The study was conducted in accordance with the Declaration of Helsinki and approved by the Institutional Review Board of Asfendiyarov Kazakh National Medical University (protocol code 873), date of approval 25 March 2020. Patient consent was waived due to the nature of the study, which involved the secondary analysis of deidentified data.

### 2.8. Statistical Analysis

Continuous variables were assessed for normality (Shapiro–Wilk test). Normally distributed continuous variables are presented as mean ± standard deviation (SD); non-normal variables as median with interquartile range (IQR). Categorical variables are summarized as counts and percentages. Differences between cord clamping groups for continuous outcomes were tested by one-way ANOVA (if normal) or Kruskal–Wallis test (if non-normal). Categorical outcomes were compared using Fisher’s exact or Chi-square test as appropriate. All statistical tests were two-tailed with significance at *p* < 0.05. All statistical analyses were performed using SAS OnDemand for Academics (version 3.81, Cary, NC, USA).

To evaluate the independent association of clamping time with maternal outcomes, we constructed multivariable regression models adjusted for age, parity and hypertension category. Because maternal blood loss (mL) was right-skewed, it was modeled with a generalized linear model (GLM) using a Gamma distribution and log link. We report effects as ratios of means (RoM) with 95% confidence intervals (CI). Hemoglobin drop and RBC drop were analyzed using ordinary least squares linear regression with heteroskedasticity-robust (HC3) standard errors; effects are presented as β coefficients (95% CI).

## 3. Results

In total, 207 women were included in the analysis. Table 1 depicts the maternal and neonatal characteristics across cord clamping groups. Maternal baseline characteristics were comparable among clamping groups in terms of age, primigravida, parity, and gestational age (*p* > 0.05). A significant difference was observed in hypertension status (*p* < 0.0001), with preeclampsia being more common in the ECC group (66.67%) and normotensive women more frequent in the DCC60s group (67.08%). Maternal blood loss did not differ significantly among groups (median 255–260 mL; *p* = 0.9128). Likewise, hemoglobin levels before and after delivery were similar (*p* > 0.05). However, RBC indices before and after delivery were different (*p* < 0.05). Systolic and diastolic blood pressures during and after childbirth were significantly higher in the ECC group compared with DCC60s and DCC120s (*p* < 0.0001). Regarding neonatal outcomes, Apgar scores at both 1 and 5 min were significantly lower in the ECC group compared with DCC60s and DCC120s (*p* = 0.014). No significant group differences were found for placental weight and infant length (*p* > 0.05), but infant weight was higher in the DCC60s group compared to other groups (*p* = 0.0013).

Table 2 summarizes maternal characteristics, including blood loss and hematologic indices, across clamping time categories. Mean (±SD) blood loss was comparable between the ECC, DCC60s, and DCC120s cord clamping groups (254.05 ± 20.29 mL, 257.34 ± 18.87 mL, and 257.60 ± 28.44 mL, respectively; *p* = 0.7760). Likewise, mean decreases in hemoglobin (Hb drop) and red blood cell counts (RBC drop) were small and did not differ significantly across groups.

Table 3 shows multivariable regression analyses adjusting for maternal age, parity, and hypertension category. The Gamma regression model for blood loss indicated no significant association between clamping time and mean blood loss after adjustment. The ratio of means (RoM) for blood loss was 0.97 (95% CI 0.93–1.01) for ECC versus DCC60s and 1.01 (95% CI 0.96–1.07) for DCC120s versus DCC60s (both *p* > 0.05). Similarly, linear models for hemoglobin and RBC changes showed no significant differences among clamping categories.

## 4. Discussion

To our knowledge, this is the first study to examine the timing of cord clamping and maternal bleeding across a broad obstetric population including hypertensive disorders in Kazakhstan. In this cohort, the timing of umbilical cord clamping had no effect on maternal blood loss or hematologic changes. Both unadjusted comparisons and multivariable-adjusted models showed that women with early versus delayed cord clamping had similar estimated blood loss, hemoglobin drop, and RBC count changes. In essence, delaying cord clamping did not increase postpartum hemorrhage in our cohort.

These findings align with prior research showing that delayed clamping does not increase maternal bleeding. A Cochrane meta-analysis of term births reported no significant differences in maternal outcomes (severe hemorrhage or hemoglobin levels) between early and late clamping [24]. De Angelis et al. conducted an RCT in term vaginal births and similarly found no significant difference in maternal hemoglobin drop between immediate and delayed clamping [25]. The consistency across these studies supports the conclusion that the timing of cord clamping has minimal impact on maternal blood loss. Our results extend this evidence to a broad Kazakh obstetric population—including women with hypertensive disorders, where preeclampsia was prevalent—confirming that delaying cord clamping up to 120 s does not increase hemorrhage. Despite postpartum hemorrhage representing a leading cause of global maternal mortality and morbidity, delayed clamping did not exacerbate it here, reinforcing its safety profile. Physiologically, this is explained by effective uterine contraction and standardized hemorrhage protocols post-placental delivery, which control bleeding independently of cord status.

Although DCC has established neonatal benefits, it may not be feasible in all deliveries. DCC can be shortened or omitted when immediate neonatal resuscitation is required or when urgent maternal stabilization is needed. In addition, during the COVID-19 pandemic, cord clamping practices varied across guidelines and clinical settings, reflecting uncertainty and infection-control constraints early in the pandemic [26,27,28]. These real-world constraints may partly explain deviations from planned DCC in some cases and should be considered when interpreting our findings.

Our results have positive implications for clinical practice. Our data support current recommendations (e.g., ACOG, WHO) that cord clamping can be safely delayed without harming the mother. For future research, larger prospective studies or multicenter trials could further examine specific subgroups (such as severe preeclampsia or placenta accreta spectrum) and assess long-term maternal recovery. It will also be valuable to explore provider-level factors (such as active management of the third stage) that might interact with clamping timing.

This study has several limitations. As an observational cross-sectional analysis, it cannot establish causality and is subject to residual confounding. We adjusted for key factors (age, parity, hypertension), but unmeasured variables (e.g., body mass index, intrapartum interventions) might influence bleeding. Finally, the classification of hypertensive disorders was based on recorded diagnosis, without gradations of severity; it is possible that severe preeclampsia could have different outcomes. Despite these caveats, the overall consistency of no difference across analyses suggests the results are robust.

## 5. Conclusions

To our knowledge, this is the first study to evaluate the association between umbilical cord clamping timing and maternal bleeding parameters in a broad obstetric cohort that included hypertensive disorders of pregnancy in Almaty, Kazakhstan. Both unadjusted analyses and multivariable models demonstrated that estimated blood loss, hemoglobin decline, and changes in red blood cell indices were similar between early and delayed clamping groups.

## Figures and Tables

**Table 1:** Characteristics of the participants based on clamping time (*N* = 207).

	ECC (*n* = 21)	DCC60s (*n* = 161)	DCC120s (*n* = 25)	*p*-Value
**Maternal feature**				
Age (years)	27 (21–38)	27 (18–39)	25 (21–36)	0.8441
Primigravida (*n*, %)				0.6177
1	9 (42.86)	46 (28.57)	5 (20)	
2	7 (33.33)	70 (43.48)	13 (52)	
3	5 (23.81)	37 (22.98)	5 (20)	
4	0	8 (4.97)	2 (8)	
Parity				0.3459
1	12 (57.14)	69 (42.86)	12 (48)	
2	7 (33.33)	65 (40.37)	11 (44)	
3	2 (9.52)	26 (16.15)	1 (4)	
4	0	1 (0.62)	1 (4)	
Hypertension				<0.0001
chronic	3 (14.29)	23 (14.29)	2 (8)	
gestational	2 (9.52)	7 (4.35)	14 (56)	
preeclampsia	14 (66.67)	23 (14.29)	8 (32)	
normotensive	2 (9.52)	108 (67.08)	1 (4)	
Pregnancy period (weeks)				0.0828
37	2 (9.52)	15 (9.32)	1 (4)	
38	4 (19.05)	26 (16.15)	9 (36)	
39	13 (61.90)	56 (34.78)	8 (32)	
40	2 (9.52)	53 (32.92)	6 (24)	
41	0	11 (6.83)	1 (4)	
Hb index (g/L)	120 (106–137)	121 (93–136)	117 (102–129)	0.1138
RBC index (×10^12^/L)	4.09 (3.65–4.55)	4.00 (3.17–4.70)	4.17 (3.76–4.59)	0.0102
Hb discharge (g/L)	116 (102–134)	118 (90–135)	114 (95–125)	0.1046
RBC discharge (×10^12^/L)	3.92 (3.50–4.50)	3.85 (3.00–4.53)	4.00 (3.24–4.40)	0.0105
Blood loss (mL)	260 (220–290)	255 (200–310)	260 (200–310)	0.9128
SBP during childbirth (mmHg)	143 (110–149)	120 (100–152)	140 (100–145)	<0.0001
DBP during childbirth (mmHg)	88 (70–94)	80 (60–95)	85 (70–90)	<0.0001
SBP after childbirth (mmHg)	130 (110–140)	115 (100–140)	125 (110–135)	<0.0001
DBP after childbirth (mmHg)	80 (70–90)	70 (60–90)	80 (70–90)	<0.0001
**Infantile features**				
Apgar score at 1 min				0.0014
<7	16 (76.19)	115 (71.43)	9 (36)	
>8	5 (23.81)	46 (28.57)	16 (64)	
Apgar score at 5 min				0.0014
<8	16 (76.19)	115 (71.43)	9 (36)	
>9	5 (23.81)	46 (28.57)	16 (64)	
Placenta weight (g)	545 (495–600)	550 (470–620)	560 (460–625)	0.1101
Weight (g)	3180 (2750–3520)	3420 (2700–4670)	3300 (2950–3680)	0.0013
Height (cm)	52 (48–55)	52 (48–56)	52 (49–56)	0.1084

ECC, early cord clamping; DCC60s, delayed cord clamping 60–119 s; DCC120s, delayed cord clamping ≥ 120 s; Hb, hemoglobin; RBC, red blood cell; SBP, systolic blood pressure; DBP, diastolic blood pressure; mL, milliliter; mmHg, millimeters of mercury; *p*-value, probability value.

**Table 2:** Maternal blood loss and hematologic indices according to clamping time category.

Variable	ECC	DCC60s	DCC120s	*p*-Value
Blood loss, mL	254.05 ± 20.29	257.34 ± 18.87	257.60 ± 28.44	0.7760
Hb drop, g/L	3.81 ± 1.29	3.73 ± 3.34	4.00 ± 3.64	0.924
RBC drop, ×10^12^/L	0.14 ± 0.06	0.17 ± 0.13	0.18 ± 0.08	0.567

ECC, early cord clamping; DCC60s, delayed cord clamping 60–119 s; DCC120s, delayed cord clamping ≥ 120 s.

**Table 3:** Adjusted associations between clamping time and maternal outcomes.

Outcome	Comparison (vs. DCC60s)	Effect Estimate	95% CI	*p*-Value
Blood loss (GLM, Gamma)	ECC vs. DCC60s	RoM = 0.97	0.93–1.01	0.1369
	DCC120s vs. DCC60s	RoM = 1.01	0.96–1.07	0.6506
Hb drop (OLS)	ECC vs. DCC60s	β = 0.54 g/L	−1.39 to 1.47	0.2542
	DCC120s vs. DCC60s	β = 1.25 g/L	−1.29 to 3.78	0.3346
RBC drop (OLS)	ECC vs. DCC60s	β = −0.002 × 10^12^/L	−0.033 to 0.029	0.9159
	DCC120s vs. DCC60s	β = 0.037 × 10^12^/L	−0.016 to 0.090	0.1742

ECC, early cord clamping; DCC60s, delayed cord clamping 60–119 s; DCC120s, delayed cord clamping ≥ 120; RoM, ratio of means.

## Data Availability

The data presented in this study are available on reasonable request from the corresponding author.

## Abbreviations

The following abbreviations are used in this manuscript:

HDP	Hypertensive disorders of pregnancy
DCC	Delayed umbilical cord clamping
ECC	Early cord clamping
DCC60s	Delayed cord clamping 60–119 s
DCC120s	Delayed cord clamping ≥ 120 s
RoM	Ratio of means
